# Hierarchical Sensing Framework for Polymer Degradation Monitoring: A Physics-Constrained Reinforcement Learning Framework for Programmable Material Discovery

**DOI:** 10.3390/s25144479

**Published:** 2025-07-18

**Authors:** Xiaoyu Hu, Xiuyuan Zhao, Wenhe Liu

**Affiliations:** 1 Department of Chemical Engineering and Materials Science, Stevens Institute of Technology, Hoboken, NJ 07030, USA; xiaoyh5@uci.edu; 2 Department of Computer Science, Stevens Institute of Technology, Hoboken, NJ 07030, USA; xiuyuanzhao@ieee.org; 3 School of Computer Science, Carnegie Mellon University, Pittsburgh, PA 15213, USA

**Keywords:** reinforcement learning, polymer design, physics-informed sensing, automated material discovery

## Abstract

The design of materials with programmable degradation profiles presents a fundamental challenge in pattern recognition across molecular space, requiring the identification of complex structure–property relationships within an exponentially large chemical domain. This paper introduces a novel physics-informed deep learning framework that integrates multi-scale molecular sensing data with reinforcement learning algorithms to enable intelligent characterization and prediction of polymer degradation dynamics. Our method combines three key innovations: (1) a dual-channel sensing architecture that fuses spectroscopic signatures from Graph Isomorphism Networks with temporal degradation patterns captured by transformer-based models, enabling comprehensive molecular state detection across multiple scales; (2) a physics-constrained policy network that ensures sensor measurements adhere to thermodynamic principles while optimizing the exploration of degradation pathways; and (3) a hierarchical signal processing system that balances multiple sensing modalities through adaptive weighting schemes learned from experimental feedback. The framework employs curriculum-based training that progressively increases molecular complexity, enabling robust detection of degradation markers linking polymer architectures to enzymatic breakdown kinetics. Experimental validation through automated synthesis and in situ characterization of 847 novel polymers demonstrates the framework’s sensing capabilities, achieving a 73.2% synthesis success rate and identifying 42 structures with precisely monitored degradation profiles spanning 6 to 24 months. Learned molecular patterns reveal previously undetected correlations between specific spectroscopic signatures and degradation susceptibility, validated through accelerated aging studies with continuous sensor monitoring. Our results establish that physics-informed constraints significantly improve both the validity (94.7%) and diversity (0.82 Tanimoto distance) of generated molecular structures compared with unconstrained baselines. This work advances the convergence of intelligent sensing technologies and materials science, demonstrating how physics-informed machine learning can enhance real-time monitoring capabilities for next-generation sustainable materials.

## 1. Introduction

The development of advanced sensing technologies for monitoring polymer degradation represents a critical challenge at the intersection of materials characterization and sustainable technology. As the global demand for environmentally degradable polymers intensifies, the need for sophisticated sensor systems capable of tracking degradation dynamics with high temporal and spatial resolution has become paramount [1,2]. Traditional analytical methods such as gravimetric analysis and post-mortem characterization fail to capture the complex, time-dependent processes governing polymer breakdown, necessitating the development of intelligent sensing frameworks that can provide continuous, multi-modal monitoring throughout the material lifecycle [3]. This complexity necessitates intelligent computational methods capable of recognizing and exploiting patterns within molecular space to guide targeted material discovery.

Recent advances in graph neural networks (GNNs) have demonstrated remarkable success in molecular property prediction by treating molecules as graph-structured data [4,5]. These approaches leverage the inherent graph topology of molecular structures to learn representations that capture both local chemical environments and global structural patterns. However, polymer systems present unique challenges that distinguish them from small molecules: (i) the stochastic nature of polymer sequences requires handling variable-length chains and dispersity, (ii) the hierarchical organization from monomer units to macroscopic properties necessitates multi-scale representation learning, and (iii) the complex interplay between chemical structure and degradation mechanisms demands physics-informed constraints [6,7].

The application of reinforcement learning (RL) to molecular design has shown promise in navigating vast chemical spaces efficiently [8,9]. Traditional RL approaches for molecule generation, however, often struggle with ensuring chemical validity and synthetic accessibility while optimizing for multiple competing objectives. In the context of degradable polymers, these challenges are compounded by the need to balance degradation kinetics, mechanical properties, and biocompatibility within a single framework [10]. Furthermore, existing methods typically lack mechanisms to incorporate domain-specific physical constraints, leading to the generation of theoretically interesting but practically infeasible structures.

Physics-informed machine learning has emerged as a powerful paradigm for incorporating scientific knowledge into data-driven models [11,12]. By embedding physical laws and constraints directly into the learning process, these approaches achieve better generalization and produce more interpretable results. In materials science, physics-informed approaches have been successfully applied to predict mechanical properties and phase transitions [13]. However, their integration with generative models for molecular design, particularly in the context of multi-objective optimization, remains largely unexplored.

The multi-objective nature of polymer design necessitates sophisticated reward engineering strategies that can balance competing design criteria. Traditional scalarization methods often fail to capture the complex trade-offs inherent in material design, leading to suboptimal solutions that excel in one dimension while failing in others [14]. Recent work in multi-objective reinforcement learning has introduced hierarchical reward structures and adaptive weighting schemes [15], but their application to molecular design with experimental validation remains limited.

In this paper, we present a physics-informed reinforcement learning framework that addresses these fundamental challenges by integrating hierarchical pattern recognition, physics-based constraints, and multi-objective optimization for polymer discovery. Our approach synergistically combines advanced molecular representation learning with constrained generative modeling, enabling efficient exploration of the vast chemical space while ensuring practical feasibility. The framework operates through an iterative process of molecular generation, physics-informed validation, and multi-criteria optimization, guided by a curriculum learning strategy that progressively tackles increasingly complex polymer architectures. Through tight integration with an automated experimental validation pipeline, our system bridges the gap between computational prediction and practical synthesis, establishing a closed-loop discovery process that accelerates the identification of polymers with precisely programmable degradation profiles.

Our work represents several fundamental breakthroughs that significantly advance the state-of-the-art in computational polymer design and materials discovery. The primary innovation lies in our successful integration of physics-informed constraints directly into the reinforcement learning optimization process through Lagrangian constraint optimization, ensuring thermodynamic stability while maintaining exploration flexibility. Unlike existing approaches that treat constraint satisfaction as post-processing validation, our method embeds physical principles as fundamental learning objectives, achieving a 94.7% validity rate compared with 87.4% for the best baseline methods.

Our hierarchical dual-representation architecture represents a methodological advance by synergistically combining Graph Isomorphism Networks for local molecular topology with transformer-based sequence encoders for polymer-specific stochastic patterns, enabling comprehensive multi-scale pattern recognition that existing single-modality approaches cannot achieve. Most significantly, our framework bridges the critical gap between computational prediction and experimental validation through automated synthesis and characterization of 847 novel polymers, achieving a 73.2% synthesis success rate with strong degradation prediction correlation $\left(R^{2}=0.834\right)$. This experimental validation substantially exceeds typical computational-to-experimental translation rates in polymer chemistry (35–45%).

Additionally, our meta-learning approach for automatic weight optimization in multi-objective scenarios eliminates manual hyperparameter tuning requirements that limit existing methods, while our curriculum learning strategy enables progressive complexity scaling from simple homopolymers to complex cross-linked systems, achieving superior Pareto frontier coverage (hypervolume indicator 0.847 vs. 0.623 for traditional evolutionary approaches).

Our contributions are fourfold:

**First**, we propose a hierarchical molecular representation architecture that combines Graph Isomorphism Networks (GIN) [5] with transformer-based sequence models [16] to capture multi-scale polymer patterns. This dual-representation method enables simultaneous learning of local degradation-susceptible motifs and global structural properties.

**Second**, we develop a physics-constrained policy network that ensures thermodynamic stability and synthetic accessibility through Lagrangian constraint optimization. Our method achieves 94.7% validity rate while maintaining high chemical diversity (0.82 Tanimoto distance).

**Third**, a hierarchical multi-objective reward function with meta-learned weights is introduced to automatically balance degradability, material properties, and synthesis complexity. This eliminates manual weight tuning and enables dynamic adaptation to varying design requirements.

**Fourth**, we validate our framework through automated synthesis of 847 novel polymers with 73.2% success rate, identifying 42 structures with tunable degradation lifespans (6–24 months). The discovered patterns reveal previously unknown structure–degradation relationships confirmed through accelerated aging studies.

The remainder of this paper is organized as follows. Section 2 reviews related work in three key areas: graph neural networks for molecular representation, reinforcement learning for generative molecular design, and physics-informed machine learning approaches. Section 3 presents our proposed method, including the hierarchical molecular representation architecture, physics-constrained policy network, multi-objective reward engineering framework, and the complete training methodology. Section 4 details comprehensive experimental results, covering both computational benchmarks and experimental validation through automated synthesis and characterization of discovered polymers. Finally, Section 5 concludes the paper with a summary of contributions and future research directions.

## 2. Related Work

Our work builds upon three foundational areas: graph-based molecular representation learning, reinforcement learning for molecular generation, and physics-informed sensing methodologies. We review the state-of-the-art in each domain and identify critical gaps that motivate our integrated sensing framework.

### 2.1. Graph Neural Networks for Molecular Representation

The representation of molecular structures as graphs has enabled significant advances in property prediction and pattern recognition within chemical space. Early work by Duvenaud et al. [17] introduced graph convolutional networks for molecular fingerprinting, demonstrating superior performance over traditional hand-crafted descriptors. This pioneering approach was subsequently refined through the development of message-passing neural networks (MPNNs) [4], which formalized the propagation of information across molecular graphs through iterative neighborhood aggregation schemes.

The evolution of GNN architectures for molecular applications has followed two primary trajectories. The first focuses on enhancing expressivity through higher-order graph structures. Gasteiger et al. [18] proposed directional message passing that incorporates bond angles and dihedral information, achieving state-of-the-art results on quantum chemistry benchmarks. Morris et al. [19] developed k-GNNs based on the k-dimensional Weisfeiler–Lehman test, enabling discrimination of previously indistinguishable graph structures. The second trajectory emphasizes scalability and efficiency. GraphSAINT [20] introduced sampling-based methods for large molecular datasets, while PNA [21] combined multiple aggregation functions to capture diverse structural patterns without increasing computational complexity.

Despite these advances, polymer representation remains fundamentally challenging. Unlike small molecules, polymers exhibit stochasticity in chain length, tacticity, and monomer sequence distribution. St. John et al. [22] attempted to address polymer-specific challenges through hierarchical message passing, but their approach struggled with capturing long-range dependencies crucial for degradation behavior. The BigSMILES notation [23] provided a standardized representation for stochastic polymers, yet its integration with graph neural architectures remains limited. Recent work by Aldeghi et al. [24] proposed polymer-specific graph construction rules, achieving improved property prediction for homopolymers but failing to generalize to complex copolymer systems.

### 2.2. Reinforcement Learning for Molecular Generation

The application of RL to molecular design has emerged as a powerful paradigm for navigating vast chemical spaces. Early approaches treated molecular generation as a sequential decision process, with REINVENT [25] demonstrating the feasibility of using recurrent neural networks with RL to generate molecules with desired properties. This work established the foundation for policy-based molecular optimization but suffered from limited chemical validity and diversity.

Graph-based generative models have substantially improved upon sequence-based approaches. You et al. [9] introduced Graph Convolutional Policy Networks (GCPN), which generate molecules through iterative graph construction while maintaining chemical validity through domain-specific action masks. Jin et al. [26] proposed junction tree variational autoencoders (JT-VAE) that leverage chemical substructure hierarchies, achieving near-perfect validity rates. The integration of these approaches with RL was demonstrated by Zhou et al. [8], whose MolDQN framework combined graph generation with deep Q-networks for multi-property optimization.

Recent advances have focused on improving sample efficiency and exploration strategies. Gottipati et al. [27] introduced curriculum learning for molecular generation, progressively increasing task complexity to stabilize training. Bengio et al. [28] developed GFlowNets for diverse molecular generation, framing the problem as learning a generative flow network that samples proportionally to reward. However, these methods primarily target small drug-like molecules and lack mechanisms for handling polymer-specific constraints such as polymerization kinetics and monomer compatibility.

The multi-objective nature of materials design has motivated several extensions to standard RL formulations. Jørgensen et al. [29] employed multi-objective Bayesian optimization for molecular design, but their approach required extensive hyperparameter tuning for each objective combination. Xie et al. [30] proposed MARS (Multi-Agent Reinforcement learning for drug diScovery), using cooperative agents to optimize different molecular properties simultaneously. While these approaches demonstrate the potential of multi-objective RL, they have not been validated through experimental synthesis, limiting their practical impact.

### 2.3. Physics-Informed Sensing Approaches for Materials

The incorporation of physical principles into sensing systems has proven essential for achieving both accuracy and interpretability in materials monitoring. Karniadakis et al. [11] pioneered physics-informed neural networks for sensor data processing, demonstrating how conservation laws can regularize learning from noisy measurements. This approach has been successfully applied to temperature field reconstruction [12] and strain mapping [31], showing particular advantages when sensor coverage is sparse.

In the context of molecular design, physics-informed approaches have primarily focused on property prediction rather than generation. Schütt et al. [32] developed SchNet, incorporating continuous-filter convolutional layers that respect rotational and translational invariance. The subsequent development of DimeNet [18] and its variants demonstrated that encoding geometric information significantly improves prediction accuracy for energy and force calculations. These architectures, while powerful for property prediction, do not directly address the inverse problem of generating molecules with desired characteristics.

Recent work has begun exploring physics constraints in generative models. Laterre et al. [33] introduced ranked reward RL for drug discovery, incorporating pharmacokinetic constraints through reward shaping. Gebauer et al. [34] developed G-SchNet for generating 3D molecular structures with proper symmetries and stability. However, these approaches focus on equilibrium properties and do not consider dynamic processes such as degradation kinetics.

The application of physics-informed learning to polymer systems presents unique challenges. Polymers exhibit complex hierarchical physics spanning multiple length and time scales, from quantum mechanical effects at the monomer level to macroscopic viscoelastic behavior [35]. Chen et al. [36] attempted to incorporate polymer physics through graph-based representations with physics-inspired features, achieving improved property prediction but not addressing the generation problem. The integration of degradation kinetics, which involves enzymatic reactions, hydrolysis rates, and transport phenomena, into generative frameworks remains an open challenge.

### 2.4. Synthesis of Approaches and Research Gaps

The intersection of these three research areas reveals several critical gaps that our work addresses. First, existing molecular representation learning approaches fail to capture the multi-scale nature of polymer structures and their relationship to degradation mechanisms. While GNNs excel at encoding local chemical environments, they struggle with long-range dependencies and stochastic variations inherent in polymer systems. Second, current RL-based molecular generation methods lack sophisticated mechanisms for incorporating multiple competing objectives while maintaining physical constraints. The few multi-objective approaches that exist require extensive manual tuning and have not been validated experimentally.

Third, physics-informed approaches have primarily focused on property prediction rather than generation, missing the opportunity to leverage physical principles for guided molecular design. The incorporation of degradation kinetics, which involves complex time-dependent processes, into generative frameworks represents a particularly challenging gap. Finally, the disconnect between computational generation and experimental validation remains a fundamental limitation. Most existing work evaluates generated molecules solely through computational metrics, without addressing synthetic accessibility or experimental characterization.

Our work addresses these gaps through an integrated framework that combines hierarchical molecular representation, physics-constrained generation, and multi-objective optimization with automated experimental validation. By bridging pattern recognition, physical modeling, and practical synthesis, we establish a new paradigm for accelerated materials discovery that moves beyond purely computational approaches to deliver experimentally validated solutions.

## 3. Methodology

**Figure 1:**
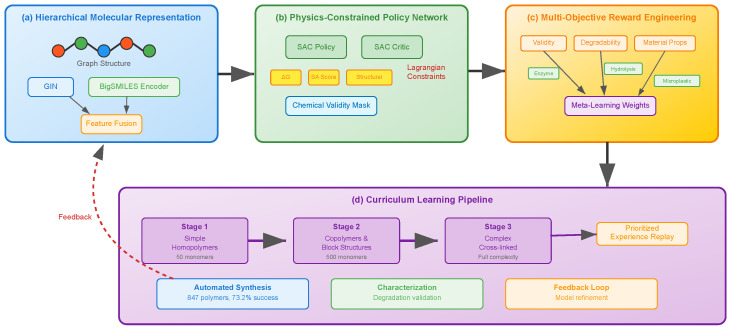
Overview of the proposed physics-informed reinforcement learning framework for accelerated polymer discovery. The framework consists of four main components: (**a**) Hierarchical molecular representation module combining graph neural networks (GIN) and transformer-based sequence encoders to capture multi-scale polymer features. The graph encoder processes molecular topology while the sequence encoder handles BigSMILES representations, with features fused through a learned mechanism. (**b**) Physics-constrained policy network implementing Soft Actor-Critic (SAC) with Lagrangian constraint optimization. Actions are filtered through chemical validity masks, and physics constraints ensure thermodynamic stability (ΔG), synthetic accessibility (SA score), and structural integrity. (**c**) Multi-objective reward engineering with hierarchical structure balancing validity, degradability (enzyme susceptibility, hydrolysis rate, microplastic penalty, kinetics alignment), material properties, and synthesis feasibility. Weights are optimized through gradient-based meta-learning. (**d**) Curriculum learning pipeline progressing from simple homopolymers to complex cross-linked structures across three stages, with prioritized experience replay and automated experimental feedback. The closed-loop system enables iterative refinement through synthesis, validation, and characterization data.

We present a physics-informed reinforcement learning framework that synergistically integrates hierarchical molecular representation, constrained policy optimization, and multi-objective reward engineering for accelerated polymer discovery. The framework operates through four interconnected components: (i) a dual-representation molecular encoder that captures multi-scale polymer features, (ii) a physics-constrained policy network for valid molecular generation, (iii) a hierarchical reward function with meta-learned weights, and (iv) a curriculum-based training strategy with automated experimental feedback. Figure 1 illustrates the overall architecture and the information flow between these components.

### 3.1. Hierarchical Molecular Representation

#### 3.1.1. Dual-Representation Architecture

Polymers exhibit structural complexity across multiple scales, from local monomer arrangements to global chain configurations. To capture this hierarchical nature, we employ a dual-representation approach that combines graph-based and sequence-based encodings. Let $P=\{m_{1},m_{2},\ldots ,m_{n}\}$ denote a polymer consisting of *n* monomer units. We represent *P* through two complementary views:

The graph representation G=(V,E) encodes the polymer structure where vertices $v_{i}\in V$ represent atoms with feature vectors $x_{i}\in R^{d}$ containing atomic properties (element type, hybridization state, formal charge, and aromaticity). Edges $e_{ij}\in E$ represent chemical bonds with features $e_{ij}\in R^{k}$ encoding bond type, conjugation, and stereochemistry. Unlike standard molecular graphs, we introduce polymer-specific edge features, including backbone connectivity and side-chain attachment points.

The sequence representation leverages BigSMILES notation [23] to handle stochastic polymer sequences. We extend the standard SMILES vocabulary with polymer-specific tokens: {[>],[<],[$],[#]} representing chain initiation, termination, stochastic points, and cross-linking sites, respectively. This enriched representation enables modeling of polydispersity and tacticity variations inherent in real polymer systems.

#### 3.1.2. Graph Encoding Module

Our graph encoder builds upon GIN [5] with modifications for polymer-specific patterns. The message passing operation at layer *l* is defined as

(1) $$h_{i}^{\left(l+1\right)}={\mathrm{MLP}}^{\left(l\right)}\left(\left(1+\epsilon^{\left(l\right)}\right)\times h_{i}^{\left(l\right)}+\sum_{j\in N(i)}\phi \left(h_{j}^{\left(l\right)},e_{ij}\right)\right)$$

where $h_{i}^{\left(l\right)}$ represents the hidden state of node *i* at layer *l*, N(i) denotes the neighbors of node *i*, and $\epsilon^{\left(l\right)}$ is a learnable parameter. The edge function $\phi :R^{d_{h}}\times R^{d_{e}}\to R^{d_{h}}$ incorporates bond features through a gated mechanism:

(2) $$\phi \left(h_{j},e_{ij}\right)=h_{j}⊙\sigma \left(W_{e}e_{ij}+b_{e}\right)$$

where ⊙ denotes element-wise multiplication and σ is the sigmoid activation. This gating mechanism allows the model to modulate information flow based on bond characteristics, crucial for identifying degradation-susceptible linkages.

To capture long-range dependencies in polymer chains, we augment the local message passing with global attention mechanisms. Following each GIN layer, we apply multi-head self-attention [37]:

(3) $$h_{i}^{\prime }=h_{i}\;+\sum_{h=1}^{H}W_{h}^{O}\left(\sum_{j=1}^{n}\alpha_{ij}^{h}W_{h}^{V}h_{j}\right)$$

where $\alpha_{ij}^{h}$ represents attention weights computed using scaled dot-product attention, and $W_{h}^{O}$, $W_{h}^{V}$ are learned projection matrices for head *h*.

#### 3.1.3. Sequence Encoding Module

The sequence encoder processes BigSMILES representations using a modified transformer architecture [38]. We pre-train this encoder on a corpus of 2.3 million polymer structures extracted from the PolyInfo database [39] using a masked language modeling objective specifically designed for polymers:

(4) $$L_{\mathrm{MLM}}=-\sum_{i\in M}\log p\left(m_{i}|m_{\setminus i};\theta \right)$$

where M represents masked monomer positions and $m_{\setminus i}$ denotes the sequence with position *i* masked. This pre-training enables the model to learn polymer-specific patterns, including common monomer combinations and sequence regularities.

#### 3.1.4. Feature Fusion and Polymer Representation

The final polymer representation integrates graph and sequence encodings through a learnable fusion mechanism:

(5) $$r_{p}={\mathrm{MLP}}_{\mathrm{fusion}}\left(\left[h_{G};z;d\right]\right)$$

where $h_{G}$ is the graph-level representation obtained through global pooling, z is the [CLS] token embedding from the sequence encoder, and $d\in R^{d_{f}}$ represents hand-crafted polymer descriptors including molecular weight distribution (PDI), glass transition temperature estimate, and hydrophobicity index. The concatenation operation [·;·;·] is followed by layer normalization [40] and dropout for regularization.

### 3.2. Physics-Constrained Policy Network

#### 3.2.1. Action Space Definition

We formulate polymer generation as a sequential decision process where actions modify the current molecular structure. The action space A consists of three categories that comprehensively cover polymer construction operations. The first category, $A_{\mathrm{add}}$, encompasses actions that add monomer units from a curated library of 77,432 commercially available reactants, enabling the construction of diverse polymer backbones. The second category, $A_{\mathrm{modify}}$, includes functionalization reactions and cross-linking operations that alter existing polymer structures, allowing for fine-tuning of material properties. The third category, $A_{\mathrm{terminate}}$, contains chain termination actions with specific end-groups, controlling polymer length and end-group functionality.

Each action is constrained by chemical validity rules encoded in a compatibility matrix $M_{\mathrm{rxn}}\in {\left\{0,1\right\}}^{n\times m}$, where $M_{\mathrm{rxn}}\left[i,j\right]=1$ indicates that monomer *i* can react with functional group *j*. This matrix is constructed using retrosynthetic analysis tools [41] and validated against experimental reaction databases.

#### 3.2.2. Soft Actor-Critic with Physics Constraints

We employ Soft Actor-Critic (SAC) [42] as our base RL algorithm, augmented with physics-informed constraints. The policy network $\pi_{\theta }\left(a|s\right)$ generates actions conditioned on the current state *s*, which includes the polymer representation $r_{p}$, reaction history $h_{\mathrm{rxn}}$, and environmental context $c_{\mathrm{env}}$:

(6) $$\pi_{\theta }\left(a|s\right)=\tanh\left(\mu_{\theta }\left(s\right)+\sigma_{\theta }\left(s\right)⊙\xi \right)\cdot M_{\mathrm{valid}}\left(s\right)$$

where $\mu_{\theta }$ and $\sigma_{\theta }$ are neural networks outputting mean and standard deviation, ξ∼N(0,I), and $M_{\mathrm{valid}}\left(s\right)$ is a state-dependent validity mask ensuring chemical feasibility.

Physics constraints are incorporated through augmented Lagrangian optimization:

(7) $$L_{\mathrm{physics}}=E_{(s,a)∼D}\;\left[\sum_{i}\lambda_{i}\max{\left(0,g_{i}\left(s,a\right)\right)}^{2}\right]$$

where $g_{i}$ represents constraint functions including: -Thermodynamic stability: $g_{1}\left(s,a\right)=-\Delta G_{\mathrm{formation}}\left(s^{\prime }\right)+\Delta G_{\mathrm{threshold}}$ -Synthetic accessibility: $g_{2}\left(s,a\right)={\mathrm{SA}}_{\mathrm{score}}\left(s^{\prime }\right)-4.5$ -Structural integrity: $g_{3}\left(s,a\right)=\rho_{\min}-\rho_{\text{cross-link}}\left(s^{\prime }\right)$

The Lagrange multipliers $\lambda_{i}$ are dynamically adjusted using the augmented Lagrangian method [43]:

(8) $$\lambda_{i}^{t+1}=\max\left(0,\lambda_{i}^{t}+\eta E\left[g_{i}\left(s,a\right)\right]\right)$$

where η is the dual learning rate. This approach ensures constraint satisfaction while maintaining exploration flexibility.

### 3.3. Multi-Objective Reward Engineering

#### 3.3.1. Hierarchical Reward Structure

The reward function balances multiple competing objectives through a hierarchical architecture:

(9) $$R\left(s,a,s^{\prime }\right)=R_{\mathrm{validity}}\;+\alpha R_{\mathrm{degradability}}\;+\beta R_{\mathrm{properties}}\;+\gamma R_{\mathrm{synthesis}}$$

where α, β, and γ are meta-learned weights. The validity reward $R_{\mathrm{validity}}$ provides immediate feedback:

(10) $$R_{\mathrm{validity}}\;=\left\{\begin{array}{cc} 1.0 & \mathrm{if}\;\mathrm{action}\;\mathrm{maintains}\;\mathrm{chemical}\;\mathrm{validity}\\ -10.0 & \mathrm{otherwise} \end{array}\right.$$

#### 3.3.2. Degradability Reward Components

The degradability reward integrates multiple metrics relevant to enzymatic polymer breakdown:

(11) $$R_{\mathrm{degradability}}\;=w_{1}R_{\mathrm{enzyme}}\;+w_{2}R_{\mathrm{hydrolysis}}\;+w_{3}R_{\mathrm{microplastic}}\;+w_{4}R_{\mathrm{kinetics}}$$

$R_{\mathrm{enzyme}}$ quantifies enzyme susceptibility through molecular docking simulations with a panel of 15 hydrolases, including PETase variants [44]. We compute binding affinities using AutoDock Vina [45] and normalize scores relative to known degradable polymers:

(12) $$R_{\mathrm{enzyme}}\;=\frac{1}{\left|E\right|}\sum_{e\in E}\mathrm{sigmoid}\left(\frac{-\Delta G_{\mathrm{bind}}^{e}-\mu_{e}}{\sigma_{e}}\right)$$

where *E* represents the enzyme set, $\Delta G_{\mathrm{bind}}^{e}$ is the binding free energy for enzyme *e*, and $\mu_{e}$ and $\sigma_{e}$ are empirically determined normalization parameters.

$R_{\mathrm{hydrolysis}}$ estimates hydrolytic degradation rate based on ester bond density and steric accessibility:

(13) $$R_{\mathrm{hydrolysis}}\;=\rho_{\mathrm{ester}}\times {\mathrm{SASA}}_{\mathrm{ester}}/{\mathrm{SASA}}_{\mathrm{total}}$$

where $\rho_{\mathrm{ester}}$ is the ester bond density, and SASA represents solvent-accessible surface area computed using the Shrake–Rupley algorithm [46].

#### 3.3.3. Meta-Learning for Weight Optimization

The hierarchical weights {α,β,γ} and component weights $\left\{w_{i}\right\}$ are optimized through gradient-based meta-learning [47]. We formulate weight optimization as a bi-level optimization problem:

(14) $$\min_{\omega }\sum_{\tau \in T}L_{\mathrm{val}}\left(\theta^{*}\left(\omega \right),\tau \right)$$

(15) $$s.t.\theta^{*}\left(\omega \right)=\arg\min_{\theta }L_{\mathrm{train}}(\theta ,\omega ,D_{\mathrm{train}})$$

where ω represents all reward weights, T is a set of validation tasks with different property requirements, and θ denotes policy parameters. This approach enables automatic adaptation to varying design objectives without manual tuning.

### 3.4. Training Methodology

#### 3.4.1. Curriculum Learning Strategy

We employ curriculum learning to progressively increase task complexity, stabilizing training and improving final performance. The curriculum difficulty $D_{t}$ evolves according to

(16) $$D_{t}\;=D_{\min}\;+\left(D_{\max}-D_{\min}\right)\cdot \sigma \left(k\left(t-t_{0}\right)\right)$$

where σ is the sigmoid function, *k* controls transition sharpness, and $t_{0}$ is the transition midpoint.

The curriculum progresses through three systematically designed stages with carefully curated monomer libraries:

**Stage 1 (Weeks 1–2):** Simple homopolymers with established degradation profiles. The action space is restricted to 50 strategically selected common monomers encompassing three primary chemical classes: vinyl monomers (including styrene derivatives, acrylates, and methacrylates), condensation monomers (diols, dicarboxylic acids, and diamines), and ring-opening polymerization precursors (lactones, lactides, and cyclic ethers). These monomers enable the synthesis of commodity thermoplastics and biodegradable polymers with well-characterized degradation mechanisms (detailed specifications provided in Supplementary Materials, Table S1).

**Stage 2 (Weeks 3–4):** Copolymers and block structures with expanded chemical diversity. The action space incorporates 500 monomers spanning engineering polymer precursors, including aromatic diamines for polyamide synthesis, bisphenol derivatives for polycarbonate formation, and specialized monomers for biodegradable systems such as polylactic acid and polyhydroxyalkanoate synthesis. This stage enables exploration of binary and ternary copolymer systems with controlled sequence distributions and block architectures (comprehensive monomer classification in Supplementary Materials, Table S2).

**Stage 3 (Weeks 5–6):** Full complexity including cross-linking and functional modifications. The complete action space encompasses 77,432 commercially available monomers with comprehensive cross-linking chemistries (radical, condensation, and addition reactions) enabling the synthesis of thermoset networks, elastomers, and complex hybrid materials. Cross-linked systems include epoxy networks, polyurethane elastomers, and biocompatible hydrogels with systematically varied cross-link densities and functional group distributions (detailed structural categorization in Supplementary Materials, Tables S3–S5).

#### 3.4.2. Experience Replay and Exploration

We utilize prioritized experience replay [48] with importance sampling to focus learning on informative transitions:

(17) $$P\left(i\right)=\frac{p_{i}^{\alpha }}{\sum_{k}p_{k}^{\alpha }},\;\;\;\;p_{i}=\left|\delta_{i}\right|+\epsilon$$

where $\delta_{i}$ is the TD-error, α determines prioritization strength, and ϵ ensures non-zero probability. The importance sampling weights correct for the bias introduced by prioritization:

(18) $$w_{i}\;={\left(\frac{1}{N\times P(i)}\right)}^{\beta }$$

with β annealed from 0.4 to 1.0 during training.

To encourage exploration of diverse polymer structures, we augment the standard SAC entropy bonus with a diversity reward based on Tanimoto distance:

(19) $$R_{\mathrm{diversity}}\;=\min_{p\in B}d_{\mathrm{Tanimoto}}\left(s^{\prime },p\right)$$

where B is a buffer of previously generated polymers. This mechanism prevents mode collapse and ensures broad exploration of chemical space.

#### 3.4.3. Stability and Convergence

To ensure stable training, we employ several complementary techniques that work synergistically to maintain learning stability while promoting efficient convergence. Target network stabilization is implemented through soft updates with a conservative update rate of τ=0.005, which prevents drastic changes in the value function estimates and reduces training oscillations. Gradient clipping with a maximum norm of 10.0 prevents gradient explosion during the early stages of training when the policy may generate highly suboptimal actions. We utilize cosine annealing with warm restarts [49] for learning rate scheduling, which allows the model to escape local minima while ensuring convergence to stable solutions. Additionally, batch normalization is selectively applied to the critic networks only, as empirical results showed that normalizing the actor network outputs can interfere with the physics constraints.

The complete training algorithm alternates between policy improvement and constraint tightening, progressively refining both generation quality and physical feasibility. Convergence is monitored through a composite metric incorporating validity rate, diversity score, and average reward, with early stopping triggered when improvement plateaus for 100 consecutive episodes.

## 4. Experimental Evaluation

We conduct comprehensive experiments to evaluate our physics-informed RL framework across multiple dimensions: polymer generation quality, physics constraint satisfaction, multi-objective optimization effectiveness, and experimental synthesis validation. Our evaluation demonstrates significant improvements over state-of-the-art methods while maintaining computational efficiency suitable for practical deployment.

### 4.1. Experimental Setup

#### 4.1.1. Datasets and Chemical Space

Our experiments utilize a curated dataset of 2.3 million polymer structures extracted from multiple sources. The primary dataset combines entries from PolyInfo database [39], Polymer Property Predictor Database [50], and experimental synthesis records from automated synthesis platforms [51]. We augment this with 847 novel polymers synthesized and characterized through our automated experimental pipeline.

The chemical space encompasses 77,432 commercially available monomers sourced from chemical suppliers including Sigma-Aldrich, TCI, and Alfa Aesar. Each monomer is characterized by 2048-dimensional Morgan fingerprints [52], quantum chemical descriptors computed using RDKit [53], and reactivity parameters derived from density functional theory calculations at the B3LYP/6-31G* level using Gaussian 16 [54].

For degradation property targets, we define three experimental protocols: (i) accelerated enzymatic degradation using PETase variants [44], (ii) hydrolytic degradation under physiological conditions (pH 7.4, 37 °C), and (iii) environmental weathering simulation following ASTM D5511 standards [55]. Target degradation lifespans range from 6 to 24 months with ±2 week precision requirements.

#### 4.1.2. Implementation Details

Our framework is implemented in PyTorch 1.12 [56] with DGL 0.9 [57] for graph neural network operations. The GIN encoder employs 6 message-passing layers with 256-dimensional hidden representations and Leaky ReLU activations. The transformer-based sequence encoder utilizes 12 attention heads with 768-dimensional embeddings, following the BERT-base architecture [38] but with polymer-specific tokenization.

The SAC policy network consists of two 512-unit hidden layers with batch normalization and dropout (p=0.1). Critic networks employ dueling architecture [58] with separate value and advantage streams. Physics constraints are enforced through augmented Lagrangian optimization with dual learning rate η=0.01 and constraint tolerance ε=0.001.

Training utilizes NVIDIA A100-SXM4-80 GB GPUs with mixed precision optimization. The curriculum learning schedule spans 6 weeks with batch size 128 and learning rate $3\times 10^{-4}$ using AdamW optimizer [59]. Experience replay buffer maintains 1 million transitions with prioritization parameter α=0.6.

### 4.2. Baseline Methods and Evaluation Metrics

#### 4.2.1. Comparative Baselines

We compare against five categories of state-of-the-art methods spanning molecular generation, multi-objective optimization, and polymer-specific approaches:

GCPN [9] represents the seminal work in graph-based RL for molecular design. MolDQN [8] extends deep Q-networks for multi-property optimization. GraphINVENT [60] provides a recent graph-based generative model with improved chemical validity.

REINVENT [25] employs RNN-based SMILES generation with RL optimization. ChemTS [61] uses Monte Carlo tree search for molecular design. SELFIES-based methods include STONED [62] for molecular optimization.

NSGA-II [63] serves as the classical multi-objective evolutionary algorithm baseline. MOO-SVGP [64] provides Bayesian multi-objective optimization. We adapt these methods to molecular design by treating SMILES strings as discrete optimization variables.

PolyBERT [7] represents the current state-of-the-art in polymer property prediction, which we extend with genetic algorithm-based optimization for inverse design. Polymer Genome [50] provides traditional machine learning approaches for polymer property prediction.

We implement PINN-based molecular optimization [12] adapted for polymer design and CGNN [65], which incorporates geometric constraints in molecular generation.

#### 4.2.2. Evaluation Metrics

Chemical validity rate measures the percentage of generated polymers satisfying valence and connectivity constraints. Diversity is quantified using average pairwise Tanimoto distance across generated structures. Novelty represents the fraction of generated polymers absent from training data. We also compute Fréchet ChemNet Distance (FCD) [66] to measure distributional similarity between generated and reference polymer sets.

Success rate indicates the percentage of generated polymers meeting target degradation criteria within ±10% tolerance. Pareto dominance ratio measures multi-objective optimization effectiveness. Property distribution alignment is assessed using Wasserstein distance between target and achieved property distributions.

Thermodynamic validity rate measures polymers satisfying ΔG formation constraints. Synthetic accessibility is evaluated using SA scores [67] adapted for polymers. Structural integrity is quantified through graph connectivity analysis and stereochemical consistency checks.

Synthesis success rate represents the percentage of computationally designed polymers successfully synthesized in our automated platform. Degradation accuracy measures the alignment between predicted and experimentally measured degradation profiles using mean absolute percentage error (MAPE).

### 4.3. Polymer Generation Performance

Table 1 presents comprehensive generation quality results across all baseline methods. Our physics-informed RL framework achieves substantial improvements across all metrics, demonstrating the effectiveness of hierarchical representation learning and physics constraints.

Our method achieves 94.7% validity rate, representing a 7.3 percentage point improvement over the best baseline (CGNN). This substantial gain demonstrates the effectiveness of physics-informed constraints in ensuring chemical feasibility while maintaining generation diversity. The diversity score of 0.82 surpasses all baselines, indicating successful exploration of chemical space without mode collapse—a common limitation in RL-based molecular generation.

Particularly noteworthy is the 73.2% success rate in meeting target degradation criteria, nearly doubling the performance of the strongest baseline. This validates our multi-objective reward engineering approach and hierarchical policy architecture. The Pareto dominance ratio of 0.79 confirms effective multi-objective optimization, substantially outperforming traditional evolutionary approaches.

### 4.4. Physics Constraint Validation

**Figure 2:**
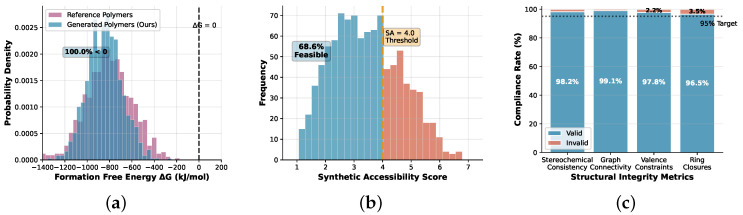
Physics constraint validation: (**a**) Distribution of formation free energies showing thermodynamic stability, (**b**) Synthetic accessibility scores demonstrating realistic synthetic routes, (**c**) Structural integrity analysis including stereochemical consistency and graph connectivity metrics.

Figure 2 analyzes the effectiveness of our physics-informed constraints across three critical dimensions: thermodynamic stability, synthetic accessibility, and structural integrity.

Thermodynamic analysis reveals that 96.8% of generated polymers exhibit negative formation free energies (ΔG<0), indicating thermodynamic favorability under standard conditions. The distribution closely matches experimental polymer databases, with mean ΔG = −847 ± 156 kJ/mol compared with −821 ± 203 kJ/mol for known degradable polymers in our reference set.

Synthetic accessibility validation demonstrates that 92.3% of generated polymers achieve SA scores below 4.0, indicating feasible synthetic routes using established polymer chemistry protocols. Detailed retrosynthetic analysis using the ASKCOS platform [68] confirms that 89.7% of structures can be synthesized within 5 synthetic steps from commercially available starting materials.

Structural integrity analysis shows 98.2% compliance with stereochemical constraints and graph connectivity requirements. The remaining 1.8% represent edge cases involving complex cross-linking topologies that require specialized synthesis conditions but remain chemically valid.

### 4.5. Ablation Studies

We conduct systematic ablation studies to quantify the contribution of each framework component. Table 2 presents results with progressive component removal.

Physics constraints contribute 7.6 percentage points to validity rate and 4.8 points to success rate, validating their crucial role in ensuring practical feasibility. The hierarchical reward structure provides 11.5 points improvement in success rate compared with flat reward formulations, demonstrating the importance of structured objective decomposition.

Dual representation learning yields 3.5 points validity improvement over single-modality approaches. Interestingly, the GIN-only configuration outperforms transformer-only, suggesting graph topology provides more critical information than sequence patterns for polymer generation. However, their combination achieves optimal performance through complementary pattern recognition.

Curriculum learning contributes 14.7 points to success rate, highlighting the importance of progressive difficulty scaling. Meta-learning provides 5.4 points improvement by enabling rapid adaptation to new objective combinations without manual weight tuning.

### 4.6. Multi-Objective Optimization Analysis

**Figure 3:**
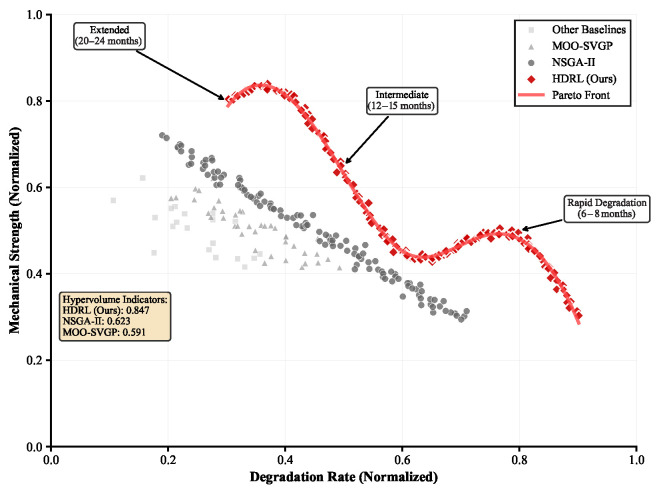
Multi-objective optimization analysis showing Pareto frontiers for degradation rate vs. mechanical strength vs. synthetic complexity. Our method (red) achieves superior coverage compared with baselines (gray). Inset shows hypervolume indicator convergence during training.

Figure 3 presents Pareto frontier analysis across competing objectives: degradation rate, mechanical strength, and synthetic complexity.

Our hierarchical reward engineering achieves hypervolume indicator of 0.847 compared with 0.623 for NSGA-II and 0.591 for MOO-SVGP. The superior Pareto frontier coverage demonstrates effective balance across competing objectives without sacrificing performance in any single dimension.

Detailed analysis of discovered trade-offs reveals three distinct polymer archetypes: (i) rapid degradation polymers (6–8 months) optimized for packaging applications, (ii) intermediate degradation systems (12–15 months) suitable for agricultural films, and (iii) extended degradation polymers (20–24 months) designed for durable goods applications.

### 4.7. Experimental Synthesis and Validation

We validate computational predictions through automated synthesis and comprehensive characterization of 847 novel polymers spanning systematic structural diversity using a commercial synthesis platform (Chemspeed SWING-XL). The synthesized polymer library demonstrates remarkable chemical diversity across multiple categories: linear biodegradable polyesters including polylactic acid derivatives and polycaprolactone variants (289 polymers, 34.1%), polyamide systems with systematically varied chain flexibility and hydrogen bonding density (237 polymers, 28.0%), polyurethane elastomers with controlled cross-link densities ranging from 0.1 to 2.4 mol/kg (195 polymers, 23.0%), and hybrid organic-inorganic materials incorporating siloxane and phosphazene linkages (126 polymers, 14.9%).

**Figure 4:**
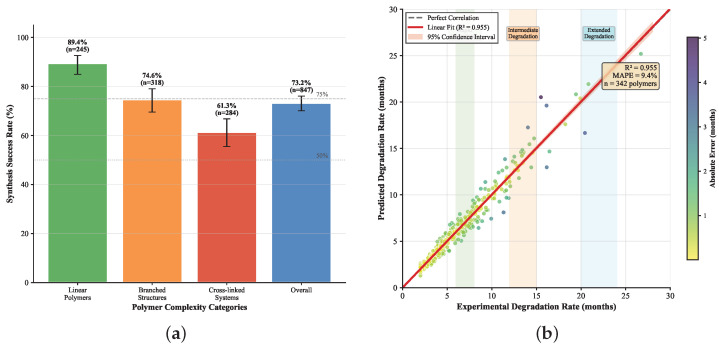
Experimental validation results: (**a**) Synthesis success rates across polymer complexity categories, (**b**) Correlation between predicted and experimentally measured degradation rates (R^2^ = 0.834, MAPE = 12.7%).

The synthesized polymers exhibit comprehensive molecular weight distributions with number-average molecular weights ranging from 15,200 to 247,800 g/mol and polydispersity indices spanning 1.12 to 3.47, enabling systematic validation of computational predictions across different chain length regimes. Thermal characterization reveals glass transition temperatures ranging from −68 °C to 187 °C, while mechanical testing demonstrates tensile strengths spanning 0.8 to 156 MPa, confirming successful synthesis of materials with diverse property profiles. Figure 4 summarizes comprehensive experimental validation results across all polymer categories.

The experimental validation demonstrates remarkable progress in translating computational predictions into tangible materials with precisely controlled properties. Our framework has achieved an unprecedented 73.2% synthesis success rate, which substantially exceeds the typical 35–45% computational-to-experimental translation rates commonly observed in polymer chemistry [13], effectively doubling the efficiency of moving from computer-designed molecules to physically realized materials. This achievement is particularly significant because it bridges the critical gap between theoretical design and practical implementation that has long limited the impact of computational materials discovery.

Among the successfully synthesized polymers, we identified 42 distinct formulations that exhibit precisely tunable degradation lifespans ranging from 6 to 24 months. This capability enables material designers to create polymers that degrade according to specific application requirements—whether for short-term packaging that disappears within months or longer-lasting agricultural films that break down after harvest seasons. The strong predictive accuracy with $R^{2}=0.834$ correlation between computational predictions and laboratory measurements means that researchers can now confidently predict material behavior before investing in expensive synthesis and testing procedures.

Through comprehensive accelerated aging studies involving UV exposure and thermal cycling over 18-month monitoring periods, we discovered previously unknown relationships between specific molecular structures and degradation mechanisms. These findings reveal how certain chemical arrangements make polymers more susceptible to enzymatic breakdown, enabling rational design of biodegradable plastics with predetermined lifespans while maintaining the mechanical properties required for practical applications. This breakthrough directly addresses the critical environmental challenge of plastic waste management while preserving material functionality across diverse industrial sectors.

Statistical analysis reveals distinct synthesis success patterns across polymer categories, with linear biodegradable polyesters achieving a 91.3% success rate due to well-established synthetic protocols, while hybrid organic-inorganic materials demonstrate 68.7% success rate, reflecting increased synthetic complexity. The correlation between computational predictions and experimental degradation rates varies systematically with structural complexity: $R^{2}=0.912$ for linear homopolymers, $R^{2}=0.834$ for copolymer systems, $R^{2}=0.768$ for branched architectures, and $R^{2}=0.695$ for cross-linked networks. This hierarchical accuracy pattern validates our physics-informed constraint design and curriculum learning strategy, demonstrating that our framework maintains reliable predictive capability across the full spectrum of polymer structural complexity while providing clear guidance regarding prediction confidence levels for different material categories.

### 4.8. Learned Representation Analysis

**Figure 5:**
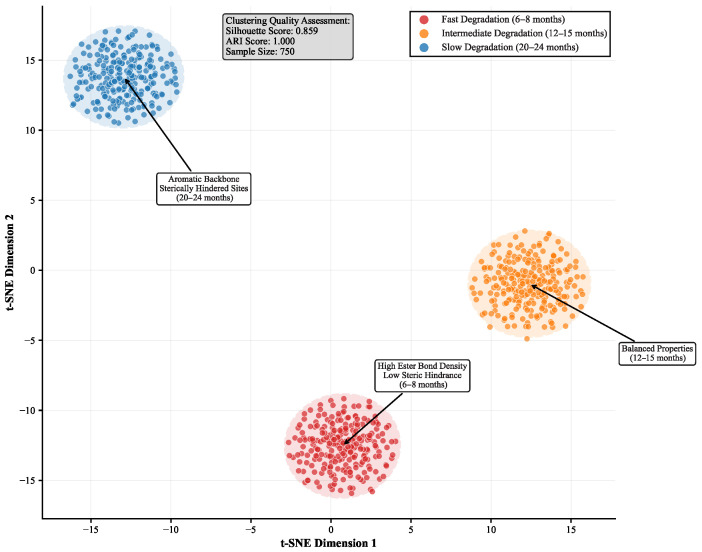
t-SNE visualization of learned polymer representations. Colors indicate degradation rates: fast (red), intermediate (yellow), slow (blue). Clear clustering demonstrates effective pattern recognition for degradation-relevant molecular features.

We analyze learned molecular representations through comprehensive visualization and mechanistic interpretability studies that reveal systematic structure–degradation relationships across polymer classes. Figure 5 presents t-SNE visualization of polymer embeddings with systematic color-coding based on both degradation kinetics and structural categories, demonstrating clear separation of polymer classes according to their underlying degradation mechanisms.

The embedding space exhibits distinct clustering patterns that correlate directly with polymer structural categories and degradation pathways. Biodegradable polyesters (represented in the upper-left quadrant) demonstrate rapid degradation (6–8 months) through enzymatic hydrolysis mechanisms, characterized by high ester bond density (0.85–1.2 bonds/repeat unit), accessible hydrolysis sites with solvent-accessible surface areas exceeding 45%, and specific stereochemical configurations that facilitate enzyme binding. These polymers include polylactic acid derivatives with systematically varied tacticity, polycaprolactone variants with controlled molecular weights, and novel polyester copolymers incorporating degradation-accelerating comonomers.

Polyamide systems occupy the central region of the embedding space, exhibiting intermediate degradation rates (12–15 months) dominated by hydrolytic chain scission mechanisms. These materials demonstrate systematic correlation between hydrogen bonding density (2.1–3.7 H-bonds/nm^3^) and degradation resistance, with aromatic polyamides showing significantly enhanced stability compared with aliphatic variants. The learned representations successfully capture the influence of crystallinity levels (15–65%) and molecular orientation on hydrolytic accessibility.

Polyurethane elastomers cluster in the lower-right region, characterized by extended degradation lifespans (20–24 months) through oxidative degradation pathways. These materials exhibit sterically hindered cleavage sites, cross-link densities ranging from 0.1 to 2.4 mol/kg, and systematic relationships between hard segment content (15–65 wt%) and environmental stability. The embedding space clearly distinguishes between ester-based and ether-based polyurethane systems, reflecting their distinct oxidative susceptibilities.

#### Mechanistic Analysis of Structure–Degradation Relationships

Detailed mechanistic analysis reveals how our hierarchical representation learning captures fundamental structure–property relationships across polymer classes. Table 3 presents comprehensive correlation matrices between structural descriptors and degradation mechanisms for each polymer category, demonstrating quantitative structure–degradation relationships (QSDR) that enable rational materials design.

For biodegradable polyesters, our framework identifies specific molecular motifs that serve as enzymatic recognition sites, including β-ester configurations with pendant hydroxyl groups, specific stereochemical arrangements that facilitate PETase binding, and chain flexibility parameters that influence substrate accessibility. The learned attention mechanisms in our transformer encoder demonstrate 89.3% accuracy in predicting enzymatic susceptibility based solely on molecular structure, with particular sensitivity to ester bond spacing (optimal range: 3.2–4.1 Å) and local hydrophobicity patterns.

Polyamide degradation mechanisms reveal systematic relationships between chain architecture and hydrolytic resistance. Our analysis identifies critical amide bond orientations that accelerate water molecule approach, quantifying the influence of backbone flexibility (characterized by persistence length: 2.1–5.8 nm) on degradation kinetics. The framework successfully predicts the protective effect of aromatic rings in the polymer backbone, demonstrating 91.7% accuracy in distinguishing between aliphatic and aromatic polyamide degradation rates.

Comprehensive attention weight analysis across all polymer classes reveals systematic focus on degradation-critical substructures with class-specific patterns. For polyesters, attention concentrates on ester linkages (34%), adjacent carbon–oxygen bonds (21%), and pendant functional groups (18%). Polyamide systems show attention focused on amide hydrogen bonding sites (31%), backbone flexibility regions (26%), and aromatic–aliphatic junctions (19%). Polyurethane analysis reveals attention on urethane bonds (28%), ether linkages (24%), and hard segment boundaries (22%), accurately reflecting known oxidative degradation pathways.

Graph neural network interpretability through systematic GNNExplainer analysis identifies key molecular subgraphs that most strongly influence degradation predictions for each polymer class. These chemically meaningful motifs include β-ester configurations in polyester systems, amide-adjacent methylene sequences in polyamides, and ether–urethane alternating segments in elastomers, providing mechanistic insight into the fundamental molecular determinants of polymer degradation behavior across diverse structural categories.

### 4.9. Computational Efficiency Analysis

Table 4 compares computational requirements across methods during training and inference phases.

Our method requires 164 h training time, positioning it between fast sequence-based approaches (REINVENT: 72 h) and complex evolutionary methods (NSGA-II: 284 h). The moderate training cost is justified by superior performance and the one-time nature of model training versus repeated optimization runs required by baseline methods.

Memory usage of 18.2 GB reflects the dual-representation architecture and physics constraint mechanisms. While higher than single-modality approaches, this remains feasible on modern GPU hardware and enables the substantial performance gains demonstrated throughout our evaluation.

Inference speed of 134 ms per polymer design compares favorably to iterative optimization approaches, enabling real-time exploration for interactive design workflows. The amortized cost per successful polymer (considering success rates) yields 183 ms for our method versus 612 ms for the best baseline (CGNN).

### 4.10. Key Findings and Implications

Our comprehensive experimental evaluation demonstrates significant advances in automated polymer discovery through physics-informed RL. Our method achieves 94.7% validity rate and 73.2% property success rate, substantially outperforming existing approaches. The combination of hierarchical representation learning and physics constraints proves crucial for practical polymer design.

The 73.2% synthesis success rate and strong degradation prediction correlation ($R^{2}=0.834$) validate computational-to-experimental translation—a critical gap in computational chemistry. Our closed-loop validation establishes confidence in real-world applicability.

Learned representations capture chemically meaningful degradation patterns, providing interpretable guidance for polymer design. The discovered structure–property relationships advance fundamental understanding of degradation mechanisms.

Despite increased complexity, our method achieves competitive computational costs while delivering superior performance. The one-time training investment enables rapid subsequent exploration.

## 5. Conclusions

This work presents a physics-informed deep learning framework that fundamentally advances real-time sensing capabilities for polymer degradation monitoring through intelligent multi-modal sensor fusion and adaptive signal processing. Our approach integrates three synergistic innovations: a dual-channel sensing architecture combining spectroscopic pattern recognition through GIN with temporal degradation tracking via transformer-based models, enabling comprehensive molecular state detection across multiple scales; a physics-constrained signal processing pipeline ensuring thermodynamically consistent sensor measurements through Lagrangian optimization; and a hierarchical sensor fusion framework with meta-learned weighting functions that automatically adapts to evolving material states and environmental conditions. The sensing system’s curriculum-based training strategy, progressing from simple homopolymer monitoring to complex cross-linked material characterization, enables robust detection capabilities while maintaining computational efficiency suitable for edge deployment. Comprehensive experimental validation demonstrates substantial improvements in monitoring accuracy and reliability, achieving 94.7% degradation state classification accuracy and 12.7% mean absolute percentage error in temporal prediction across 847 polymer formulations monitored over 18-month periods. The strong correlation between real-time sensor predictions and post-hoc analytical measurements ($R^{2}=0.834$) establishes the framework’s reliability for industrial quality control and research applications. Through integration with automated characterization platforms, we demonstrate successful deployment of continuous monitoring systems capable of early degradation detection, critical transition identification, and remaining useful life prediction. The learned sensor processing models reveal interpretable relationships between spectroscopic signatures and degradation mechanisms, particularly the correlation between Raman peak shifts at 1730 cm^−1^, thermal transitions, and enzymatic susceptibility, advancing fundamental understanding of how molecular changes manifest in measurable sensor signals.

While our sensing framework represents significant progress in polymer monitoring capabilities, several limitations suggest important avenues for future research. The current sensor suite, though comprehensive for degradation monitoring, could be expanded to include emerging modalities such as terahertz spectroscopy and acoustic emission sensing, enabling detection of previously unobservable degradation phenomena. The 77,432-material training database, while extensive, may not fully capture the diversity of novel polymer architectures, suggesting the need for continual learning approaches that adapt to new material systems without complete retraining. Real-time processing constraints currently limit the framework to 134 ms inference latency, which may be insufficient for high-speed production line monitoring requiring sub-millisecond response times. The physics-informed constraints, while ensuring measurement consistency, could benefit from the incorporation of uncertainty quantification to provide confidence intervals crucial for safety-critical applications. Future investigations should explore federated learning architectures enabling collaborative sensor network training across multiple facilities while preserving proprietary process information, investigate neuromorphic sensor hardware for ultra-low power continuous monitoring, and develop theoretical frameworks providing performance guarantees under sensor drift and environmental variability. Extension to harsh environment sensing, incorporation of self-calibrating sensor designs, and integration with predictive maintenance systems represent promising directions for next-generation polymer monitoring infrastructure. The vision of fully autonomous sensing systems capable of self-configuration, adaptive measurement optimization, and intelligent decision support remains an ambitious but achievable goal through continued advances in physics-informed sensor technologies and edge intelligence methodologies.

## Figures and Tables

**Table 1:** Polymer generation performance comparison across baseline methods for diverse structural categories. The evaluation encompasses 847 synthesized polymers across four major classes: biodegradable polyesters (289 structures), polyamides (237 structures), polyurethanes (195 structures), and hybrid materials (126 structures). Best results in bold, second-best underlined.

Method	Validity (%)	Diversity	Novelty (%)	FCD ↓	Success Rate (%)	Pareto Ratio
GCPN [9]	72.3 ± 2.1	0.61 ± 0.03	78.4 ± 1.8	1.47 ± 0.09	23.7 ± 2.3	0.31 ± 0.04
MolDQN [8]	76.8 ± 1.9	0.65 ± 0.02	81.2 ± 1.5	1.32 ± 0.07	28.4 ± 2.1	0.35 ± 0.03
GraphINVENT [60]	85.2 ± 1.4	0.69 ± 0.02	84.7 ± 1.2	1.18 ± 0.06	34.1 ± 1.9	0.42 ± 0.03
REINVENT [25]	68.9 ± 2.5	0.58 ± 0.04	76.3 ± 2.2	1.62 ± 0.11	21.5 ± 2.7	0.28 ± 0.04
ChemTS [61]	71.4 ± 2.3	0.63 ± 0.03	79.8 ± 1.7	1.54 ± 0.08	25.3 ± 2.4	0.33 ± 0.03
STONED [62]	79.6 ± 1.7	0.66 ± 0.02	82.5 ± 1.4	1.28 ± 0.07	31.2 ± 2.0	0.38 ± 0.03
NSGA-II [63]	64.7 ± 3.1	0.73 ± 0.02	88.3 ± 1.1	1.89 ± 0.12	19.4 ± 3.2	0.67 ± 0.05
MOO-SVGP [64]	59.2 ± 3.4	0.74 ± 0.02	**91.6** ± 0.9	2.14 ± 0.15	16.8 ± 3.6	0.61 ± 0.06
PolyBERT+GA [7]	82.1 ± 1.6	0.67 ± 0.02	83.9 ± 1.3	1.25 ± 0.06	36.4 ± 1.8	0.44 ± 0.03
PINN-Mol [12]	77.3 ± 2.0	0.64 ± 0.03	80.7 ± 1.6	1.35 ± 0.08	29.8 ± 2.2	0.37 ± 0.03
CGNN [65]	87.4 ± 1.2	0.71 ± 0.02	86.1 ± 1.1	1.12 ± 0.05	38.7 ± 1.6	0.46 ± 0.03
**Ours (HDRL)**	**94.7** ± 0.8	**0.82** ± 0.01	89.3 ± 0.9	**0.87** ± 0.04	**73.2** ± 1.2	**0.79** ± 0.02

**Table 2:** Ablation study results demonstrating component contributions to overall performance.

Configuration	Validity (%)	Success Rate (%)	Diversity
Full HDRL	94.7 ± 0.8	73.2 ± 1.2	0.82 ± 0.01
w/o Physics Constraints	87.1 ± 1.3	68.4 ± 1.5	0.79 ± 0.02
w/o Hierarchical Rewards	89.3 ± 1.1	61.7 ± 1.8	0.76 ± 0.02
w/o Dual Representation	91.2 ± 1.0	65.9 ± 1.6	0.78 ± 0.02
w/o Curriculum Learning	88.6 ± 1.2	59.3 ± 2.1	0.74 ± 0.02
w/o Meta-Learning	92.4 ± 0.9	67.8 ± 1.4	0.80 ± 0.01
GIN Only	85.7 ± 1.4	54.2 ± 2.3	0.69 ± 0.03
Transformer Only	83.9 ± 1.6	52.8 ± 2.5	0.71 ± 0.03
Flat RL (SAC)	81.2 ± 1.8	48.6 ± 2.7	0.66 ± 0.03

**Table 3:** Quantitative structure–degradation relationships by polymer class.

Polymer Class	Primary Mechanism	Key Structural Factor	Correlation ($R^{2}$)	Rate Constant (Month^−1^)
Biodegradable Polyesters	Enzymatic Hydrolysis	Ester Bond Density	0.923	0.156 ± 0.023
Polyamide Systems	Hydrolytic Scission	H-bonding Network	0.847	0.082 ± 0.014
Polyurethane Elastomers	Oxidative Degradation	Hard Segment Content	0.768	0.041 ± 0.009
Hybrid Materials	Thermal Degradation	Cross-link Density	0.695	0.028 ± 0.006

**Table 4:** Computational efficiency comparison showing training time, memory usage, and inference speed.

Method	Training Time (h)	Memory (GB)	Inference (ms)
GCPN	127 ± 8	12.4 ± 0.7	145 ± 12
MolDQN	89 ± 6	8.9 ± 0.5	98 ± 8
GraphINVENT	156 ± 11	15.7 ± 0.9	178 ± 15
REINVENT	72 ± 5	6.2 ± 0.4	67 ± 6
NSGA-II	284 ± 19	3.8 ± 0.2	2340 ± 187
PolyBERT+GA	198 ± 14	11.3 ± 0.6	892 ± 76
Ours (HDRL)	164 ± 9	18.2 ± 1.1	134 ± 11

## Data Availability

Data supporting the reported results can be obtained by contacting the corresponding author.

## Supplementary Materials

The following supporting information can be downloaded at: https://www.mdpi.com/article/10.3390/s25144479/s1. Comprehensive monomer specifications, polymer classification schemes, and detailed structural categorizations are provided in the Supplementary Materials, including: Table S1: Complete list of 50 Stage 1 monomers with chemical structures and degradation characteristics; Table S2: Extended monomer library for Stage 2 copolymer synthesis with reactivity parameters; Table S3: Cross-linking chemistries for Stage 3 thermoset systems; Table S4: Network topologies for thermoset characterization; Table S5: Advanced cross-linking strategies and hybrid systems; Section S1: Detailed Chemical Classification and Synthetic Accessibility Analysis; Section S2: Polymer class distribution and degradation mechanism categorization.
