# Supplementary material for: Hierarchical Sensing Framework for Polymer Degradation Monitoring: A Physics-Constrained Reinforcement Learning Framework for Programmable Material Discovery
Source: Sensors (Basel). 2025 Jul 18;25(14):4479. doi: 10.3390/s25144479 (PMC12298545; doi:10.3390/s25144479)
Supplement: Supplementary file 1 [file sensors-25-04479-s001.zip › sensors-3727344-supplementary.pdf]

# Supplementary Materials
# Hierarchical Sensing Framework for Polymer Degradation Monitoring: A Physics-Constrained Reinforcement Learning Framework for Programmable Material Discovery

Xiaoyu Hu[1,*], Xiuyuan Zhao[2], Wenhe Liu[3]

## Contents

**1 Section S1: Detailed Chemical Classification and Synthetic Accessibility Analysis 2**
1.1 Chemical Space Categorization . . . 2
1.1.1 Polymerization Mechanism Classification . . . 2
1.1.2 Structural Feature Analysis . . . 2
1.2 Synthetic Accessibility Assessment . . . 2

**2 Section S2: Polymer Class Distribution and Degradation Mechanism Categorization 3**
2.1 Experimental Polymer Distribution . . . 3
2.1.1 Biodegradable Polyesters (289 polymers, 34.1%) . . . 3
2.1.2 Polyamide Systems (237 polymers, 28.0%) . . . 3
2.1.3 Polyurethane Elastomers (195 polymers, 23.0%) . . . 3
2.1.4 Hybrid Organic-Inorganic Materials (126 polymers, 14.9%) . . . 3
2.2 Degradation Pathway Analysis . . . 4
2.2.1 Enzymatic Degradation Pathways . . . 4
2.2.2 Hydrolytic Degradation Mechanisms . . . 4
2.2.3 Oxidative Degradation Pathways . . . 4

# 1 Section S1: Detailed Chemical Classification and Synthetic Accessibility Analysis

## 1.1 Chemical Space Categorization

The 77,432 commercially available monomers in our database are systematically classified according to polymerization mechanisms and structural features. The classification scheme enables targeted curriculum learning and ensures comprehensive coverage of polymer chemical space.

### 1.1.1 Polymerization Mechanism Classification

**Chain-Growth Polymerization (45,823 monomers, 59.1%):**

- Vinyl monomers: 23,456 structures including acrylates, methacrylates, styrene derivatives, and vinyl halides
- Diene monomers: 8,934 structures for elastomer synthesis including butadiene, isoprene, and chloroprene derivatives
- Ring-opening monomers: 13,433 structures including lactones, lactams, cyclic ethers, and cyclic carbonates

**Step-Growth Polymerization (31,609 monomers, 40.9%):**

- Difunctional monomers: 18,765 structures including diols, dicarboxylic acids, diamines, and diisocyanates
- Polyfunctional monomers: 12,844 structures for cross-linked networks including triols, tetracarboxylic acids, and polyamines

### 1.1.2 Structural Feature Analysis

Chemical diversity is quantified using Morgan fingerprints with radius 3 and 2048 bits. The Tanimoto distance matrix reveals distinct clustering patterns corresponding to chemical families. Average pairwise distances range from 0.23 (within vinyl monomer subclasses) to 0.89 (between aromatic polyamide precursors and aliphatic polyesters).

## 1.2 Synthetic Accessibility Assessment

Synthetic accessibility scores are computed using a modified SA score algorithm adapted for polymer precursors. The assessment incorporates:

- Fragment contribution analysis based on 1.2M reaction precedents from the Reaxys database
- Retrosynthetic complexity estimation using 15 reaction types common in polymer chemistry
- Commercial availability scoring from 47 chemical suppliers worldwide
- Regulatory and safety constraints for industrial-scale synthesis

Distribution of SA scores across the complete monomer database:

- Excellent accessibility ($SA < 2.0$): 34,567 monomers (44.6%)
- Good accessibility ($2.0 \leq SA < 3.0$): 28,445 monomers (36.7%)
- Moderate accessibility ($3.0 \leq SA < 4.0$): 11,234 monomers (14.5%)
- Challenging synthesis ($SA \geq 4.0$): 3,186 monomers (4.1%)

# 2 Section S2: Polymer Class Distribution and Degradation Mechanism Categorization

## 2.1 Experimental Polymer Distribution

The 847 successfully synthesized polymers span four major structural categories with systematic property variations:

### 2.1.1 Biodegradable Polyesters (289 polymers, 34.1%)

Primary degradation mechanism: Enzymatic hydrolysis catalyzed by esterases and lipases

Structural subcategories:

- Polylactic acid derivatives (89 polymers): L-lactide, D-lactide, and racemic mixtures with tacticity control
- Polycaprolactone variants (76 polymers): Molecular weights 15,000-180,000 g/mol with controlled end-groups
- Polyhydroxyalkanoates (67 polymers): PHB, PHV, and copolymers with varied side-chain length
- Novel aliphatic polyesters (57 polymers): Succinate, adipate, and sebacate-based systems

Degradation kinetics: First-order with rate constants 0.089-0.234 month$^{-1}$ (pH 7.4, 37°C)

### 2.1.2 Polyamide Systems (237 polymers, 28.0%)

Primary degradation mechanism: Hydrolytic chain scission with hydrogen bonding modulation

Structural subcategories:

- Aliphatic polyamides (134 polymers): Nylon 6, 66, 610, and novel sequences with 4-12 methylene units
- Aromatic polyamides (68 polymers): Terephthalamide and isophthalamide-based systems
- Bio-based polyamides (35 polymers): Derived from renewable dicarboxylic acids and diamines

Degradation kinetics: Complex multi-phase with initial rates 0.034-0.127 month$^{-1}$

### 2.1.3 Polyurethane Elastomers (195 polymers, 23.0%)

Primary degradation mechanism: Oxidative degradation via free radical processes

Structural subcategories:

- Polyester-urethanes (89 polymers): Adipate and caprolactone-based soft segments
- Polyether-urethanes (76 polymers): PPO and PTMO-based systems with varied molecular weights
- Hybrid polyurethanes (30 polymers): Mixed ester-ether soft segments with novel architectures

Cross-link density range: 0.08-2.73 mol/kg with systematic mechanical property correlations

### 2.1.4 Hybrid Organic-Inorganic Materials (126 polymers, 14.9%)

Primary degradation mechanism: Thermal degradation with selective bond cleavage

Structural subcategories:

- Siloxane-organic hybrids (67 polymers): PDMS-polyurethane and PDMS-polyacrylate systems
- Phosphazene-based polymers (34 polymers): Polyphosphazenes with varied side-chain functionality
- Metal-organic frameworks (25 polymers): Coordination polymers with degradable organic linkers

## 2.2 Degradation Pathway Analysis

Comprehensive mechanistic analysis reveals distinct degradation pathways for each polymer class with quantitative structure-activity relationships.

### 2.2.1 Enzymatic Degradation Pathways

For polyester systems, enzyme-substrate interactions follow Michaelis-Menten kinetics:

$$v = \frac{V_{\max}[S]}{K_m + [S]} \tag{1}$$

where $V_{\max}$ ranges from 0.23-1.87 $\mu$ mol/min/mg enzyme and $K_m$ values span 12-156 $\mu M$ depending on polymer structure and enzyme type.

Key structural determinants of enzymatic susceptibility:

- Ester bond spacing: Optimal range 3.2-4.1 Å for PETase binding
- Crystallinity: Inverse correlation with degradation rate ($R^2 = 0.78$)
- Surface hydrophobicity: Critical threshold at contact angle 72°

### 2.2.2 Hydrolytic Degradation Mechanisms

Polyamide hydrolysis follows pseudo-first-order kinetics with pH dependence:

$$k_{obs} = k_0 + k_H[H^+] + k_{OH}[OH^-] \tag{2}$$

Rate constants at pH 7.4 and 37°C:

- Aliphatic polyamides: $k_0$ = 2.3-8.7 × $10^{-8}$ $s^{-1}$
- Aromatic polyamides: $k_0$ = 0.8-3.2 × $10^{-8}$ $s^{-1}$

### 2.2.3 Oxidative Degradation Pathways

Polyurethane oxidation involves multiple competing pathways:

- Ether bond cleavage: Activation energy 87-124 kJ/mol
- Urethane bond dissociation: Activation energy 156-203 kJ/mol
- Cross-linking reactions: Temperature-dependent above 120°C

Table S1: Complete list of 50 Stage 1 monomers with chemical structures and degradation characteristics

| Monomer ID | Chemical Name | Structure (SMILES) | MW (g/mol) | SA Score | Degradation Rate |
|---|---|---|---|---|---|
| M001 | L-Lactide | O1C(C)C(=O)OC(C)C1=O | 144.13 | 1.2 | Fast |
| M002 | D-Lactide | O1C(C)C(=O)OC(C)C1=O | 144.13 | 1.2 | Fast |
| M003 | $\varepsilon$ -Caprolactone | O=C1CCCCCO1 | 114.14 | 1.1 | Fast |
| M004 | $\beta$-Butyrolactone | O=C1CCCO1 | 86.09 | 1.3 | Fast |
| M005 | Methyl acrylate | C=CC(=O)OC | 86.09 | 1.0 | Moderate |
| M006 | Ethyl acrylate | C=CC(=O)OCC | 100.12 | 1.0 | Moderate |
| M007 | Methyl methacrylate | C=C(C)C(=O)OC | 100.12 | 1.0 | Moderate |
| M008 | Styrene | C=Cc1ccccc1 | 104.15 | 1.1 | Slow |
| M009 | Vinyl acetate | C=COC(=O)C | 86.09 | 1.0 | Fast |
| M010 | Ethylene glycol | OCCO | 62.07 | 1.0 | Fast |
| M011 | 1,4-Butanediol | OCCCCO | 90.12 | 1.1 | Fast |
| M012 | Adipic acid | OC(=O)CCCCC(=O)O | 146.14 | 1.2 | Fast |
| M013 | Succinic acid | OC(=O)CCC(=O)O | 118.09 | 1.1 | Fast |
| M014 | Hexamethylene diamine | NCCCCCCN | 116.20 | 1.3 | Moderate |
| M015 | Sebacic acid | OC(=O)CCCCCCCCC(=O)O | 202.25 | 1.4 | Fast |
| M016 | Terephthalic acid | OC(=O)c1ccc(cc1)C(=O)O | 166.13 | 1.5 | Slow |
| M017 | Isophthalic acid | OC(=O)c1cccc(c1)C(=O)O | 166.13 | 1.5 | Slow |
| M018 | 4,4'-Methylenedianiline | Nc1ccc(cc1)Cc2ccc(cc2)N | 198.26 | 2.1 | Moderate |
| M019 | Ethylene oxide | C1CO1 | 44.05 | 1.0 | Fast |
| M020 | Propylene oxide | CC1CO1 | 58.08 | 1.0 | Fast |
| M021 | Glycolide | O=C1COC(=O)CO1 | 116.07 | 1.3 | Fast |
| M022 | Trimethylene carbonate | O=C1OCCCO1 | 102.09 | 1.4 | Fast |
| M023 | $\beta$-Propiolactone | O=C1CCO1 | 72.06 | 1.5 | Fast |
| M024 | $\delta$-Valerolactone | O=C1CCCCO1 | 100.12 | 1.2 | Fast |
| M025 | 3-Hydroxybutyric acid | CC(O)CC(=O)O | 104.10 | 1.3 | Fast |
| M026 | 3-Hydroxyvaleric acid | CCC(O)CC(=O)O | 118.13 | 1.4 | Fast |
| M027 | Butyl acrylate | C=CC(=O)OCCCC | 128.17 | 1.1 | Moderate |
| M028 | 2-Ethylhexyl acrylate | C=CC(=O)OCC(CC)CCCC | 184.28 | 1.3 | Moderate |
| M029 | Hydroxyethyl methacrylate | C=C(C)C(=O)OCCO | 130.14 | 1.2 | Fast |
| M030 | N-Vinyl pyrrolidone | C=CN1CCCC1=O | 111.14 | 1.4 | Moderate |
| M031 | Acrylamide | C=CC(=O)N | 71.08 | 1.0 | Fast |
| M032 | Methacrylamide | C=C(C)C(=O)N | 85.11 | 1.1 | Fast |
| M033 | 1,6-Hexanediol | OCCCCCCO | 118.17 | 1.2 | Fast |
| M034 | 1,4-Cyclohexanediol | OC1CCC(O)CC1 | 116.16 | 1.3 | Fast |
| M035 | Bisphenol A | CC(C)(c1ccc(O)cc1)c2ccc(O)cc2 | 228.29 | 1.8 | Slow |

Table 1: Table S1 (continued)

| Monomer ID | Chemical Name | Structure (SMILES) | MW (g/mol) | SA Score | Degradation Rate |
|---|---|---|---|---|---|
| M036 | 4,4'-Dihydroxydiphenyl sulfone | Oc1ccc(cc1)S(=O)(=O)c2ccc(O)cc2 | 250.27 | 2.0 | Slow |
| M037 | Pentaerythritol | OCC(CO)(CO)CO | 136.15 | 1.5 | Fast |
| M038 | Trimethylolpropane | CCC(CO)(CO)CO | 134.17 | 1.4 | Fast |
| M039 | Phthalic anhydride | O=C1OC(=O)c2ccccc12 | 148.12 | 1.3 | Moderate |
| M040 | Maleic anhydride | O=C1OC(=O)C=C1 | 98.06 | 1.2 | Fast |
| M041 | Itaconic acid | C=C(C)C(=O)O | 130.10 | 1.3 | Fast |
| M042 | Fumaric acid | OC(=O)C=CC(=O)O | 116.07 | 1.2 | Fast |
| M043 | Glutaric acid | OC(=O)CCCC(=O)O | 132.12 | 1.2 | Fast |
| M044 | Pimellic acid | OC(=O)CCCCCC(=O)O | 160.17 | 1.3 | Fast |
| M045 | Azelaic acid | OC(=O)CCCCCCCC(=O)O | 188.22 | 1.4 | Fast |
| M046 | Dodecanedioic acid | OC(=O)CCCCCCCCCCC(=O)O | 230.30 | 1.6 | Fast |
| M047 | 1,5-Pentanediamine | NCCCCCN | 102.18 | 1.4 | Moderate |
| M048 | 1,10-Decanediamine | NCCCCCCCCCCN | 172.31 | 1.7 | Moderate |
| M049 | Isophorone diamine | CC1(C)CC(N)CC(C)(CN)C1 | 170.30 | 2.2 | Moderate |
| M050 | 4,4'-Methylenedi(cyclohexylamine) | NC1CCC(CC1)CC2CCC(N)CC2 | 210.36 | 2.4 | Moderate |

Table S2: Extended monomer library for Stage 2 copolymer synthesis with reactivity parameters

| Category | Monomer Name | Reactivity Ratio | Q-value | e-value | Class |
|---|---|---|---|---|---|
| Vinyl Monomers | Methyl acrylate | $r_1$=0.42, $r_2$=1.95 | 0.60 | 0.60 | Acrylate |
| | Ethyl acrylate | $r_1$=0.38, $r_2$=2.05 | 0.58 | 0.58 | Acrylate |
| | Butyl acrylate | $r_1$=0.35,$r_2$=2.15 | 0.56 | 0.56 | Acrylate |
| | 2-Ethylhexyl acrylate | $r_1$=0.33, $r_2$=2.25 | 0.54 | 0.54 | Acrylate |
| | Methyl methacrylate | $r_1$=1.85, $r_2$=0.37 | 0.74 | 0.40 | Methacrylate |
| | Ethyl methacrylate | $r_1$=1.82, $r_2$=0.39 | 0.72 | 0.38 | Methacrylate |
| | Butyl methacrylate | $r_1$=1.78, $r_2$=0.42 | 0.70 | 0.36 | Methacrylate |
| | Styrene | $r_1$=0.52, $r_2$=0.46 | 1.00 | -0.80 | Aromatic |
| | $\alpha$-Methylstyrene | $r_1$=0.25, $r_2$=0.33 | 0.98 | -0.72 | Aromatic |
| | 4-Methylstyrene | $r_1$=0.48, $r_2$=0.51 | 1.02 | -0.75 | Aromatic |
| | Vinyl acetate | $r_1$=0.01, $r_2$=55.0 | 0.026 | -0.22 | Vinyl ester |
| | Vinyl chloride | $r_1$=1.68, $r_2$=0.23 | 0.044 | 0.20 | Vinyl halide |
| | Vinylidene chloride | $r_1$=3.2, $r_2$=0.30 | 0.36 | 0.36 | Vinyl halide |
| | Acrylonitrile | $r_1$=1.20, $r_2$=0.04 | 0.60 | 1.20 | Nitrile |
| | Methacrylonitrile | $r_1$=1.12, $r_2$=0.15 | 0.81 | 0.81 | Nitrile |
| Engineering Monomers | 4,4'-Oxydiphthalic anhydride | - | - | - | Anhydride |
| | 3,3',4,4'-Biphenyltetracarboxylic dianhydride | - | - | - | Anhydride |
| | Pyromellitic dianhydride | - | - | - | Anhydride |
| | 4,4'-Hexafluoroisopropylidene diphthalic anhydride | - | - | - | Anhydride |
| | 4,4'-Oxydianiline | - | - | - | Aromatic diamine |
| | 3,4'-Oxydianiline | - | - | - | Aromatic diamine |
| | 4,4'-Methylenedianiline | - | - | - | Aromatic diamine |
| | 2,4-Diaminotoluene | - | - | - | Aromatic diamine |

Table 2: Table S2 (continued)

| Category | Monomer Name | Reactivity Ratio | Q-value | e-value | Class |
|---|---|---|---|---|---|
| Bio-based Monomers | 1,3-Phenylene diamine | - | - | - | Aromatic diamine |
| | 1,4-Phenylene diamine | - | - | - | Aromatic diamine |
| | 4,4'-Diaminodiphenyl sulfone | - | - | - | Aromatic diamine |
| | 3,3'-Diaminodiphenyl sulfone | - | - | - | Aromatic diamine |
| | Levulinic acid | - | - | - | Bio-carboxylic |
| | Itaconic acid | $r_1$=0.21, $r_2$=2.3 | 0.37 | 0.77 | Bio-unsaturated |
| | Muconic acid | - | - | - | Bio-diacid |
| | 2,5-Furandicarboxylic acid | - | - | - | Furan derivative |
| | Vanillic acid | - | - | - | Lignin derivative |
| | Protocatechuic acid | - | - | - | Lignin derivative |
| | 1,4-Butanediol (bio) | - | - | - | Bio-diol |
| | 1,5-Pentanediamine (bio) | - | - | - | Bio-diamine |
| Specialty Monomers | Acrylic acid | $r_1$=1.30, $r_2$=0.50 | 0.77 | 0.77 | Carboxylic acid |
| | Methacrylic acid | $r_1$=2.40, $r_2$=0.65 | 0.65 | 0.65 | Carboxylic acid |
| | 2-Hydroxyethyl acrylate | $r_1$=0.41, $r_2$=1.98 | 0.59 | 0.59 | Hydroxyl functional |
| | 2-Hydroxyethyl methacrylate | $r_1$=1.80, $r_2$=0.38 | 0.73 | 0.39 | Hydroxyl functional |
| | Glycidyl methacrylate | $r_1$=1.75, $r_2$=0.40 | 0.71 | 0.37 | Epoxy functional |
| | Allyl glycidyl ether | $r_1$=0.02, $r_2$=0.5 | 0.03 | -1.05 | Epoxy functional |
| | 3-(Trimethoxysilyl)propyl methacrylate | $r_1$=1.73, $r_2$=0.43 | 0.69 | 0.35 | Silane functional |
| | Vinyl triethoxysilane | $r_1$=0.01, $r_2$=45 | 0.025 | -0.25 | Silane functional |
| | N,N-Dimethylacrylamide | $r_1$=1.35, $r_2$=0.44 | 0.89 | -0.35 | Amide functional |

Table 2: Table S2 (continued)

| **Category** | **Monomer Name** | **Reactivity Ratio** | **Q-value** | **e-value** | **Class** |
|---|---|---|---|---|---|
| | N-Isopropylacrylamide | $r_1$=1.32, $r_2$=0.46 | 0.87 | -0.33 | Amide functional |

Table S3: Cross-linking chemistries for Stage 3 thermoset systems

| **Cross-link Type** | **Chemistry** | **Activation Energy (kJ/mol)** | **Reaction Conditions** | **Network Type** |
|---|---|---|---|---|
| Epoxy-Amine | Ring opening addition | 65-85 | RT-150°C, 2-24h | Thermoset |
| Epoxy-Anhydride | Esterification | 75-95 | 120-180°C, 1-8h | Thermoset |
| Urethane Formation | Isocyanate + Hydroxyl | 45-65 | RT-80°C, 0.5-4h | Elastomer |
| Urea Formation | Isocyanate + Amine | 55-75 | RT-120°C, 1-6h | Rigid foam |
| Acrylate Polymerization | Free radical | 85-125 | UV or 60-120°C | Coating |
| Methacrylate Polymerization | Free radical | 75-115 | UV or 70-140°C | Dental |
| Thiol-Ene | Radical addition | 35-55 | UV, RT-80°C | Optical |
| Thiol-Epoxy | Nucleophilic addition | 25-45 | RT-100°C, base cat. | Adhesive |
| Click Chemistry | Azide-Alkyne | 15-35 | RT-60°C, Cu catalyst | Biomedical |
| Michael Addition | Nucleophile-Acceptor | 40-60 | RT-80°C, basic pH | Hydrogel |

Table S4: Network topologies for thermoset characterization

| **Network Parameter** | **Measurement Method** | **Typical Range** | **Property Impact** | **Degradation Effect** |
|---|---|---|---|---|
| Cross-link Density | Dynamic mechanical analysis | 0.1-5.0 mol/kg | Modulus, Brittleness | Restricts access |
| Mesh Size | Swelling experiments | 0.5-50 nm | Permeability | Controls diffusion |
| Glass Transition | DSC/DMA | -80 to 250°C | Service temperature | Mobility dependent |
| Gel Fraction | Extraction analysis | 65-98% | Network completeness | Soluble fraction |
| Cross-link Efficiency | NMR/FTIR | 40-95% | Mechanical properties | Defect sites |
| Network Heterogeneity | Small angle scattering | 1.2-4.5 | Stress distribution | Preferential degradation |
| Chain Length | SEC analysis | 500-5000 g/mol | Flexibility | End-group effects |
| Functionality | Chemical analysis | 2.1-6.0 | Network connectivity | Branch points |

Table S5: Advanced cross-linking strategies and hybrid systems

| **Hybrid System** | **Component A** | **Component B** | **Synergistic Effect** | **Application** |
|---|---|---|---|---|
| Dual-cure Systems | UV acrylate | Thermal epoxy | Sequential curing | Rapid prototyping |
| IPN Networks | Polyurethane | Epoxy resin | Enhanced toughness | Aerospace |
| Semi-IPN | Cross-linked PU | Linear polymer | Damping properties | Vibration control |
| Gradient Networks | Variable cross-link | Functional gradient | Stress distribution | Biomedical |
| Self-healing | Reversible bonds | Healing agents | Damage recovery | Coatings |
| Shape memory | Thermoplastic | Cross-linked phase | Programmable shape | Smart materials |
| Nanocomposite | Organic matrix | Inorganic nanoparticles | Enhanced properties | Structural |
| Bio-hybrid | Synthetic polymer | Biological component | Biocompatibility | Tissue engineering |
| Degradable Networks | Stable cross-links | Labile linkages | Controlled degradation | Drug delivery |
| Photo-responsive | Light-stable matrix | Photolabile cross-links | Remote control | Actuators |
